# Safety assessment of *Streptococcus thermophilus* IDCC 2201 used for product manufacturing in Korea

**DOI:** 10.1002/fsn3.1925

**Published:** 2020-10-02

**Authors:** O‐Hyun Ban, Sangki Oh, Chanmi Park, Won Yeong Bang, Bo Som Lee, Soo‐Yeon Yang, Seung A Chae, Young Hoon Jung, Jungwoo Yang

**Affiliations:** ^1^ Ildong Bioscience Gyeonggi‐do South Korea; ^2^ School of Food Science and Biotechnology Kyungpook National University Daegu South Korea; ^3^ Institute of Fermentation Biotechnology Kyungpook National University Daegu South Korea

**Keywords:** Antibiotic resistance, Probiotics, Safety evaluation, Single‐dose oral toxicity, *Streptococcusthermophilus*

## Abstract

Safety evaluation of probiotics has become increasingly important for human consumption in food industry. The aims of this study were to assess safety of *Streptococcus thermophilus* IDCC 2201 through in vitro and in vivo tests. In results, this strain was found to be negative for hemolytic and β‐glucuronidase activity. In addition, *thermophilus* IDCC 2201 was susceptible to nine antibiotics suggested by EFSA. In accordance with MIC tests, whole‐genome analysis indicated that *S*.* thermophilus* IDCC 2201 neither harbors antibiotic resistance nor toxigenic genes. Furthermore, none of the biogenic amines including tyramine and histamine was produced and negligible amounts of D‐lactate were produced by *S*.* thermophilus* IDCC 2201. Finally, it was confirmed that there was no mortality and toxicity throughout single‐dose oral toxicity tests in rats. Therefore, we report that *S. thermophilus* IDCC 2201 is considered to be safe for human consumption as probiotics.

## Graphical abstract

The safety assessment of *S*.* thermophilus* IDCC 2201 used for probiotics manufacturing in Ildong Bioscience was performed through in vitro and in vivo tests. The strain was susceptible to nine antibiotics by MICs test and did not have any antibiotic resistance gene as analyzed in the whole‐genome sequencing. We report that *S*.* thermophilus* IDCC 2201 is considered to be safe for human consumption as probiotics.

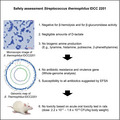

## 1 INTRODUCTION

Lactic acid bacteria have been considered safe and commonly used in foods and fermentation processes (Bernardeau et al., 2006; Zielinska & Kolonzyn‐Krajewska, 2018). Typically, they are Gram‐positive, mostly nonmotile, nonspore‐forming, facultative anaerobic (or microaerophilic), and rod‐ (or cocci‐) shaped bacteria that are utilized in fermented dairy and nondairy products such as fermented vegetables, meats, and beverages (Nuraida, 2015). Although the majority of probiotics contain *Lactobacillus* and *Bifidobacterium*, *Streptococcus* genus is one of the most widely used probiotic strain.

*Streptococcus thermophilus* has been generally isolated from traditional fermented milk and yogurt products (Vendramin et al., 2017; Zhang et al., 2019). In particular, *S*.* thermophilus* has been used as a starter for dairy products because of rapid acidifying capacity during fermentation process (Naumenko et al., 2019; Santos et al., 2019). It has been also reported to exhibit several health‐beneficial effects such as growth inhibition of many pathogenic bacteria and strong adherence to the gastrointestinal tract (Braun et al., 2020; Iyer et al., 2010; Wu et al., 2014).

Although probiotics have been regarded as GRAS (generally recognized as safe) strains, recent significant developments in the field of probiotics have brought their safety aspects into focus (Sanders et al., 2010). Thus, the FAO/WHO guidelines recommend performing clinically standardized methods for assessing the safety of probiotics (Hill et al., 2014). Specifically, to ensure that probiotics are safe for human and animal consumption, the guidelines recommend establishing the antibiotic resistance patterns of probiotic strains and the absence of acquired or transferable resistance factors (Yahav et al., 2018). In accordance with this notion, the transmission of antibiotic resistance genes from probiotics to gut microbiota is a major health concern (Wang et al., 2006).

In this study, the safety of *S. thermophilus* IDCC 2201 used as a part of commercial probiotic products was evaluated by using phenotypic and genotypic methods. Firstly, in vitro tests were performed to analyze its hemolytic and enzymatic activities, and the minimal inhibitory concentrations (MICs) against a variety of antibiotics. Secondly, whole‐genome analysis was performed to determine whether this strain harbors toxigenic and antibiotic resistance genes and whether these genes are transferable to commensal or pathogenic bacteria. Finally, a single‐dose oral toxicity test was performed in rats. Therefore, this study verifies the safety of *S. thermophilus* IDCC 2201.

## 2 MATERIALS AND METHODS

### 2.1 Bacterial strains and growth conditions

*Streptococcus thermophilus* IDCC 2201 strain isolated from home‐made yogurt was identified phenotypically and genotypically and have been included in products manufactured by Ildong Bioscience, Korea (Table S1 and Table S2). It was grown in MRS medium (BD Difco, Franklin Lakes, NJ, USA) at 37°C with 0.5% CO_2_ in static incubator. *Staphylococcus aureus* ATCC 25923 was used as a positive strain for hemolysis assay, and it was incubated in brain heart infusion (BHI; BD Difco) medium at 37°C with vigorous shaking at 200 rpm.

### 2.2 Hemolysis assay and enzymatic activities test

Hemolysis assay was performed by streaking bacterial cells on sheep blood agar plates (BBL Microbiology Systems, Cockeysville, MD, USA). Then, the plates were incubated at 37°C until the clear zones around the colony were observed.

Enzymatic activities were determined using an API‐ZYM kit (BIOMÉRIUX, Marcy‐l'Étoile, France) according to the manufacturer's instructions. Briefly, cell cultures were harvested and resuspended in sterile distilled water. Afterward, 65 μl suspension of McFarland standard was deposited into each well, and the plates were incubated at 37°C for 4 hr. Then, one drop of ZYM‐A and ZYM‐B reagent was added to each well, and enzyme activity was analyzed after 5 min.

### 2.3 Determination of minimum inhibitory concentration and whole‐genome analysis

The MICs of *S*.* thermophilus* IDCC 2201 were determined by Etest method (Mayrhofer et al., 2008). Briefly, 1% (v/v) of cells grown overnight was transferred into fresh MRS broth. When the cell density of the culture plates reached approx. at 1–2 × 10^8^ CFU (colony forming units)/ml, cells were spread onto MRS agar plate prior to the overlay of antibiotic strips (Liofilchem, Roseto degli Abruzzi, Italy). The antibiotics tested in the study were ampicillin, vancomycin, gentamycin, kanamycin, streptomycin, erythromycin, clindamycin, tetracycline, and chloramphenicol according to the technical guidelines of the EFSA (EFSA, 2012).

Whole‐genome sequencing of *S*.* thermophilus* IDCC 2201 was performed by LAS (Gochon, Gimpo, Korea). Virulence factors and antibiotic resistance genes were searched using the VFDB database (Chen et al., 2005) and ResFinder program with database (Zankari et al., 2012), respectively. BLASTN parameters were set to identity of >80% and coverage of >60% for identification of target antibiotics: aminoglycoside, beta‐lactam, colistin, fosfomycin, fusidic acid, macrolide, nitroimidazole, oxazolidinone, phenicol, quinolone, rifampicin, sulfonamide, tetracycline, trimethoprim, and glycopeptides. Transposases and conjugal transfer proteins were annotated using the BLASTP against the transposases and conjugal transfer proteins retrieved from NCBI GenBank. Prophage regions were identified using PHASTER web‐based program (Arndt et al., 2016).

### 2.4 Determination of biogenic amine concentrations

The biogenic amines (BAs) produced by *S*.* thermophilus* IDCC 2201 were determined according to the previous study (Deepika Priyadarshani & Rakshit, 2011) and EFSA standard protocol (EFSA, 2012) with minor modifications. Briefly, a single colony was cultured overnight in 10 ml of MRS broth at 37°C in static incubator. The grown cells were transferred into 10 ml of fresh MRS broth with a dilution at 1:100 and incubated at 37°C for 24 hr. Then, the supernatant from the culture was collected by centrifugation at 2,300 ×*g* for 5 min at 4°C. Prior to the quantification of BAs, 0.75 ml of supernatant was mixed with the equivalent of 0.1 M HCl and filtered with 0.45 µm membrane to extract BAs. For the derivatization, 1 ml of the extracted BAs was incubated in a 70°C water bath for 10 min, followed by addition with 200 µl of saturated NaHCO_3_, 20 µl of 2 M NaOH, and 0.5 ml of dansyl chloride (10 mg/ml acetone). The derivatized BAs were then mixed with 200 µl of proline (100 mg/ml H_2_O) and incubated in the absence of light at room temperature for 15 min. The mixture was made up to 5 ml with acetonitrile (HPLC grade; Sigma‐Aldrich, St. Louis, MO, USA). Finally, BAs were separated and quantified by high performance liquid chromatography (HPLC; LC‐NETII/ADC, Jasco, Macclesfield, UK) equipped with an Athena C18 column (4.6 mm × 250 mm, ANPEL Inc, Yexie Town, Songjiang District Shanghai, China). Aqueous acetonitrile solution (67:33 of H_2_O, v/v) was used as a mobile phase at a constant flow rate of 0.8 ml/min. Peaks were detected at 254 nm by using a UV detector (UV‐2075 plus, Jasco) and quantified according to calibration curves of each BA such as tyramine, histamine, putrescine, 2‐phenethylamine, and cadaverine.

### 2.5 L/D‐lactate formation

The quantification of L‐lactate and D‐lactate was performed by using an assay kit (Megazyme, Bray, Ireland). Briefly, cell‐free supernatant from overnight culture of *S*.* thermophilus* IDCC 2201 was assayed with the following enzymes; L‐/D‐lactate dehydrogenase and glutamate‐pyruvate transaminase. Then, the absorbance of diluted supernatant was measured at 340 nm, and L‐/D‐lactate concentrations were calculated according to the manufacturer's protocol.

### 2.6 Acute oral toxicity test

Acute oral toxicity (AOT) test was performed by Korea Testing and Research Institute (KTR; Hwasun‐gun, Jeollanam‐do, Korea). The AOT test was performed according to OECD guidelines (2008) for testing of chemicals. Briefly, 12 Crl:CD(*SD*) female rats aged 9–10 weeks were divided into four groups of 3 rats each. Each group was orally dosed with 300 or 2,000 mg of freeze‐dried *Streptococcus* powder in 10 ml sterilized water per kg body weight. Then, the mortality, signs of toxicity, and body weight changes were monitored for 14 days. Finally, 100 ml of isoflurane was injected to euthanize the rats, and an autopsy for the examination of organs was performed on the 14th day.

## 3 RESULTS AND DISCUSSION

### 3.1 Hemolytic property and enzymatic activities

A hemolytic activity is generally caused by hemolysin produced by bacteria and it induces the lysis of red blood cells that result in mild to severe infection by a variety of pathogens (Nodzo et al., 2014). Thus, hemolytic activity is an important criterion of safety in selecting probiotics (Sorokulova et al., 2008). In this study, *S*.* thermophilus* IDCC 2201 were found to be negative for hemolytic activity. In contrast, *Staphylococcus aureus* ATCC 25923 as a positive control clearly induced a β‐hemolysis on sheep blood agar (Figure S1).

β‐Glucosidase of lactic acid bacteria hydrolyzes glucose conjugates from plants, generating a variety of plant secondary metabolites in the colon. These resulting metabolites function as health‐promoting substances (e.g., antioxidants) (Michlmayr et al., 2013). However, it was also reported to produce potential toxins (e.g., deoxynivalenol) or carcinogenic compounds in rare cases (Cole & Fuller, 1987). *Streptococcus thermophilus* IDCC 2201 tested in this study was found to have no activity of β‐glucosidase (Table 1 and Figure S2). Another safety concern is that β‐glucuronidase produced by microorganisms can develop toxic steroidal (e.g., estrogen) or carcinogenic compounds and thereby increase risk for colorectal cancer (Kim & Jin, 2001). Preferably, *S. thermophilus* IDCC 2201 in this study have no activity of β‐glucuronidase (Table 1 and Figure S2). Thus, *S. thermophilus* IDCC 2201 is unlikely to produce toxic chemicals during the fermentation.

**Table 1:** Enzymatic activities of *Streptococcus thermophilus* IDCC 2201

Enzyme	*S*.* thermophilus* IDCC 2201
Alkaline phosphate	−
Esterase	++
Esterase Lipase	−
Lipase	−
Leucine arylamidase	+++
Valine arylamidase	++
Cystine arylamidase	+
Trypsin	−
α‐chymotrypsin	−
Acid phosphatase	++
Naphthol‐AS‐BI‐phosphohydrolase	+
α‐galactosidase	−
β‐galactosidase	+++
β‐glucuronidase	−
α‐glucosidase	−
β‐glucosidase	−
*N*‐acetyl‐β‐glucosaminidase	−
α‐mannosidase	+
α‐fucosidase	−

### 3.2 Determination of MICs and whole‐genome analysis

*Streptococcus thermophilus* IDCC 2201 were evaluated whether they are susceptible to a variety of antibiotics, which are typically used to treat enterococcal infections (EFSA, 2012). In this test, nine antibiotics were used as follows: ampicillin, vancomycin, gentamicin, kanamycin, streptomycin, erythromycin, clindamycin, tetracycline, and chloramphenicol. In results, *S*.* thermophilus* IDCC 2201 was susceptible to all the antibiotics tested (Table 2). In accordance with MIC tests, the whole‐genome analysis of *S. thermophilus* IDCC 2201 (1.79 Mb) indicated that it has no gene or similar gene characterized as antibiotic resistance gene was found in this genome (Figure S3). In previous study (Rizzotti et al., 2009), *S. thermophilus* isolated from Italian soft cheeses was resistant to tetracycline and harbored the genes *tet*(S), *tet*(M), and *tet*(L). Meanwhile, gene coding for hyaluronic acid capsule as a virulence factor was found with the BLASTP parameters in this genome. However, this factor is frequently found in the genomes of many other *S. thermophilus* strains (Wessels et al., 1991). Additionally, 81 mobile elements such as transposase were found in this genome.

**Table 2:** Minimal inhibitory concentration against a variety of antibiotics

	AMP	VAN	GEN	KAN	STR	ERY	CLI	TET	CHL
Cutoff value (µg/ml)^a^	2	4	32	*n*.r.	64	2	2	4	4
*Streptococcus thermophilus* IDCC 2201	0.064/S^b^	0.025/S	16/S	96	32/S	0.094/S	0.023/S	0.019/S	1.5/S

Abbreviations: AMP, ampicillin; CHL, chloramphenicol; CLI, clindamycin; ERY, erythromycin; GEN, gentamycin; KAN, kanamycin; *n*.r.: not required; STR, streptomycin; TET, tetracycline; VAN, vancomycin.

a EFSA (European Food Safety Authority), 2012. *EFSA Journal*, 16(3), 5206–5224.

b S, Susceptible

### 3.3 Biogenic amine production

Biogenic amines (BAs) are organic compounds with low molecular weight and can be produced by lactic acid bacteria harboring amino acid decarboxylase gene (e.g., *tdc*) (Barbieri et al., 2019). They are present in various fermented foods and have a lot of biological activities such as essential psychoactive or vasoactive effects (Erdag et al., 2018). However, some of these amines are so bioactive that they can cause various adverse effects in human health (Spano et al., 2010). For example, they can affect the vascular system as well as the central nervous system, resulting in cardiovascular hypertension, vomiting, and headache. Furthermore, some BAs have the potential to be converted into powerful carcinogens (e.g., nitrosamine) (Lonvaur‐Funel, 2001). Among BAs, histamine and tyramine are considered the most important in food safety, and they are responsible for scombroid fish poisoning (histamine intoxication), food‐induced migraine, and hypertensive crisis. (Izquierdo‐Pulido et al., 1996). Putrescine has been implicated in cell proliferation and has been linked to cancer. In this study, tyramine, histamine, putrescine, 2‐phenethylamine, and cadaverine were not detected in *S*.* thermophilus* IDCC 2201 (Table 3). Thus, it was concluded that *S. thermophilus* IDCC 2201 is considered safe in terms of BAs production.

**Table 3:** Biogenic amine production

	Tyramine	Histamine	Putrescine	Cadaverine	2‐Phenethylamine
*S*.* thermophilus* IDCC 2201	n.d.	n.d.	n.d.	n.d.	n.d.

n.d.: not detected.

### 3.4 L/D‐lactate formation

Lactate can be produced either via homofermentative or heterofermentative pathway by lactic acid bacteria (Drinan et al., 1976). Although L‐lactate is predominantly produced from pyruvate by L‐lactate dehydrogenase, D‐lactate can be produced by lactic acid bacteria, depending on the strains and environmental conditions (Zuniga et al., 1993). D‐lactate is so difficult to be metabolized that it can be accumulated in humans, causing D‐lactic acidosis (Petersen, 2005; Schiraldi et al., 2003). Therefore, D‐lactate formation by lactic acid bacteria is also an important criterion for safety evaluations. In this study, 20.1 g/L (99.85%) of L‐lactate and 0.03 g/L (0.15%) of D‐lactate were produced by *S. thermophilus* IDCC 2201 (Table 4). Thus, the results indicate that the formation of D‐lactate by this strain is negligible.

**Table 4:** D‐lactate production

Strains	L‐Lactate (g/L)	D‐Lactate (g/L)	Ratio (%)	
			L‐form	D‐form
*Streptococcus thermophilus* IDCC 2201	20.15 ± 0.41	0.03 ± 0.00	99.85	0.15

### 3.5 Single‐dose acute oral toxicity study

To evaluate the safety of *S. thermophilus* IDCC 2201 in vivo, a single‐dose acute oral toxicity tests were performed with 4 test groups (Table 5). The observation for 14 days indicated that a single oral dose of 2.3 × 10^11^–1.6 × 10^12^ CFU and 2.2 × 10^11^–1.6 × 10^12^ CFU of *S*.* thermophilus* IDCC 2201 did not cause mortality and toxicity in 9 week‐aged rats and 10 week‐aged rats, respectively. Additionally, there were no significant changes in the appearances (e.g., skin, fur, and eye), behaviors (e.g., tremors, convulsion, and salivation), body weight (Table 5), and feed intake of the rats. Any gross pathological change was not found in all rats throughout the autopsy. Thus, we conclude that there is no evidence of any toxicity in the rats received *S. thermophilus* IDCC 2201.

**Table 5:** Change in body weight of the tested rats

Group	Dosage (g/kg BW^a^)	Day after administration				
		0	1	3	7	14
9 week‐aged	St^b^,300	211.0 ± 8.7	236.2 ± 9.1	242.0 ± 6.9	255.5 ± 8.5	262.3 ± 6.6
	St,2000	212.7 ± 12.1	232.4 ± 4.1	236.7 ± 10.6	244.8 ± 8.8	255.7 ± 11.1
10 week‐aged	St,300	236.4 ± 8.0	266.4 ± 6.3	268.3 ± 6.6	275.5 ± 13.8	284.7 ± 11.4
	St,2000	224.6 ± 15.0	239.1 ± 19.3	250.0 ± 16.3	257.3 ± 22.5	261.4 ± 26.6

a BW, body weight.

b St, *Streptococcus thermophilus* IDCC 2201.

## 4 CONCLUSION

The safety assessment of *S*.* thermophilus* IDCC 2201 used for probiotics manufacturing in Ildong Bioscience was performed through in vitro and in vivo tests. In results, this strain was found to be negative for hemolytic activity, and no endogenous enzymes which make toxic substances were found. In addition, the strain was susceptible to nine antibiotics by MICs test and did not have any antibiotic resistance gene as analyzed in the whole‐genome sequencing. Additionally, biogenic amines were not produced and D‐lactate formation was negligible after fermentation. In acute oral toxicity test, no hazardous phenomenon was observed in rats. Therefore, we report that *S. thermophilus* IDCC 2201 is considered to be safe for human consumption as probiotics. Finally, these findings contribute to screening for safe potential probiotics and for safe starters in the dairy industries.

## CONFLICT OF INTEREST

The authors declare no conflict of interest.

## ETHICAL STATEMENT

The animal experiments in this study were conducted by Korea Testing and Research Institute (KTR; Hwasun‐gun, Jeollanam‐do, Korea) under Animal protection act (no. 14651) and laboratory animal act (no. 15278) by Korea government.

## Supporting information

Supplementary Material
