# Supplementary material for: Safety assessment of Streptococcus thermophilus IDCC 2201 used for product manufacturing in Korea
Source: Food Sci Nutr. 2020 Oct 2;8(11):6269–74. doi: 10.1002/fsn3.1925 (PMC7684611; doi:10.1002/fsn3.1925)
Supplement: Supplementary file 1 — Supplementary Material [file FSN3-8-6269-s001.doc]

**SUPPLEMETARY INFORMATION**

**Safety assessment of *Streptococcus thermophilus* IDCC 2201 used for product manufacturing in Korea**

O-Hyun Ban^a^, Sangki Oh^a^, Chanmi Park^a^, Won Yeong Bang^b^, Bo Som Lee^b^, Soo-yeon Yang^a^, Seung A Chae^a^, Young Hoon Jung^b^*, Jungwoo Yang^a^*

^a^Ildong BioScience, 17 Poseunggongdan-ro, Pyeongtaek-si, Gyeonggi-do, 17957, Republic of Korea

^b^School of Food Science and Biotechnology, Kyungpook National University, Daegu, 41566, Republic of Korea

*Correspondence: Tel.: +82-31-646-3180; Fax: +82-70-7500-2592

E-mail: [younghoonjung@knu.ac.kr](mailto:younghoonjung@knu.ac.kr) (YH Jung) & [yjw@ildong.com](mailto:yjw@ildong.com) (J. Yang)


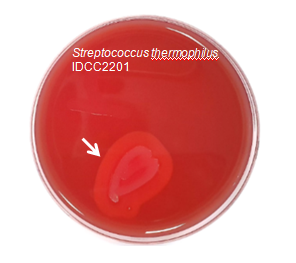


**Supplementary Figure 1.** Hemolytic activities of *S. thermophilus* IDCC 2201. As a positive control for β-hemolysis, *Staphylococcus aureus* ATCC 25923 was used (arrows indicated) and figures are representatives from 3 independent experiments.


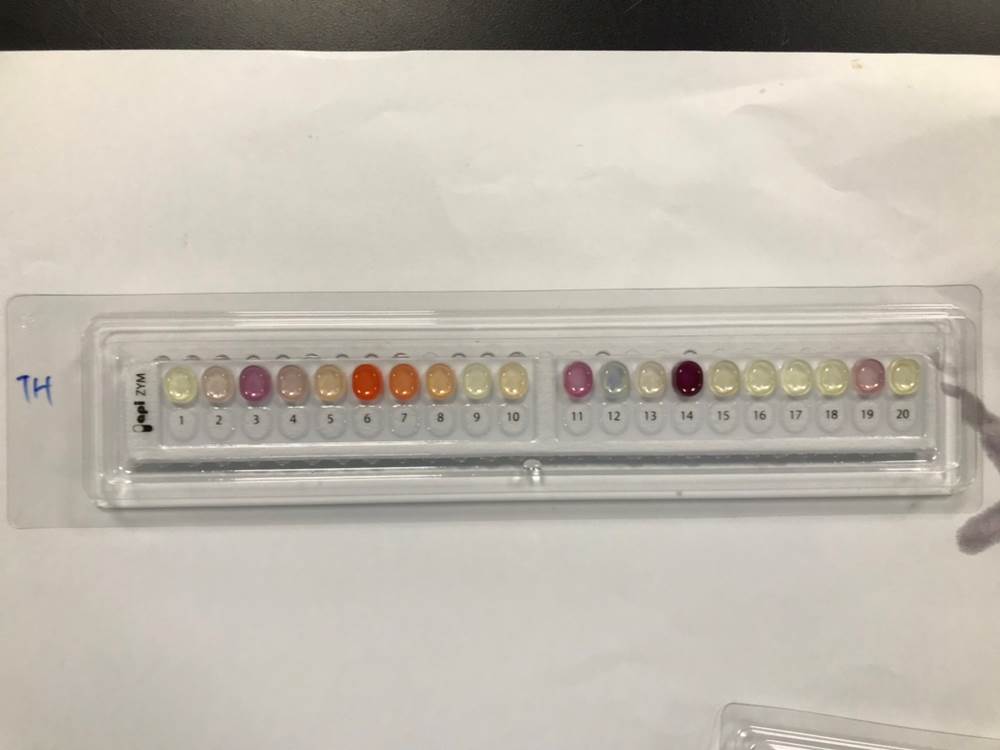


**Supplementary Figure 2.** Enzymatic activities of *S. thermophilus* IDCC 2201.


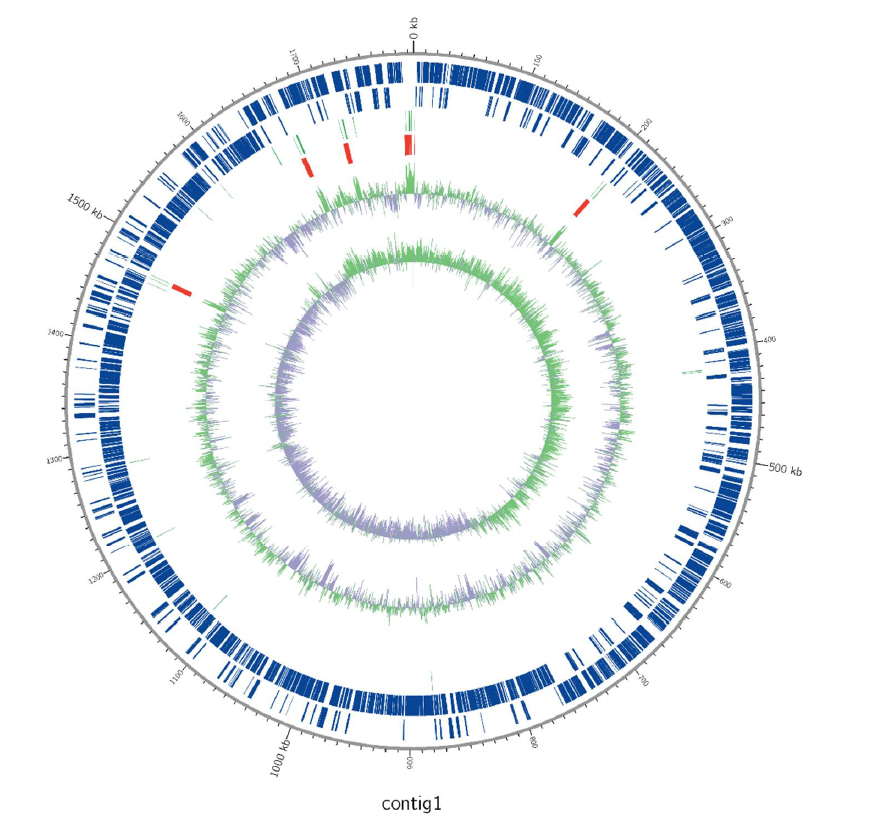


**Supplementary Figure 3.** Genomic map of *S. thermophilus* IDCC 2201. Marked characteristics are shown from outside to the center; CDS on forward strand, CDS on reverse strand, tRNA, rRNA, GC content and GC skew.

**Supplementary Table 1.** Taxonomic information acquired by 16s rDNA sequencing (A) and summary of the genome (B).

A

| Property | Term |
| --- | --- |
| Classification | Domain: *Bacteria* |
|  | Phylum: *Firmicutes* |
|  | Class: *Bacilli* |
|  | Order: *Lactobacillales* |
|  | Family: *Streptococcaceae* |
|  | Genus: *Streptococcus* |
|  | Species: *Streptococcus thermophilus* |

B

| Property | Statistics |
| --- | --- |
| Total genome size | 1,794,693 bp |
| GC ratio | 39.19% |
| No. of contigs | 1 |
| No. of CDSs | 1,905 |
| No. of rRNA genes | 18 |
| No. of rRNA genes | 67 |

| **No** | **Substrate** | **Result** | **No** | **Substrate** | **Result** | **No** | **Substrate** | **Result** | **No** | **Substrate** | **Result** | **No** | **Substrate** | **Result** |
| --- | --- | --- | --- | --- | --- | --- | --- | --- | --- | --- | --- | --- | --- | --- |
| 1 | Glycerol | - | 11 | D-Glucose | + | 21 | α-Methyl-D-glucoside | - | 31 | Sucrose | + | 41 | D-Lyxose | - |
| 2 | Erythritol | - | 12 | D-Fructose | - | 22 | N-Acethyl-Glucosamine | - | 32 | Trehalose | + | 42 | D-Tagatose | - |
| 3 | D-Arabinose | - | 13 | D-Mannose | + | 23 | Amygdaline | - | 33 | Inuline | - | 43 | D-Fucose | - |
| 4 | L-Arabinose | - | 14 | L-Sorbose | - | 24 | Arbutine | - | 34 | Melizitose | - | 44 | L-Fucose | - |
| 5 | Ribose | - | 15 | Rhamnose | - | 25 | Esculine | + | 35 | D-Raffinose | - | 45 | D-Arabitol | - |
| 6 | D-Xylose | - | 16 | Dulcitol | - | 26 | Salicine | - | 36 | Amidon | - | 46 | L-Arabitol | - |
| 7 | L-Xilose | - | 17 | Inositiol | - | 27 | Cellobiose | - | 37 | Glycogene | - | 47 | Gluconate | - |
| 8 | Adonitol | - | 18 | Mannitol | - | 28 | Maltose | - | 38 | Xylitol | - | 48 | 2-keto-gluconate | - |
| 9 | β-Methyl-xylose | - | 19 | Sorbitol | - | 29 | Lactose | + | 39 | Gentibiose | - | 49 | 5-keto-gluconate | - |
| 10 | Galactose | +^w^ | 20 | α-Methyl-D-mannoside | - | 30 | Melibiose | - | 40 | D-Turanose | - |  |  |  |

**Supplementary Table 2.** Carbohydrate utilization by *S. thermophilus* IDCC 2201.
